# Deep learning enabled fast 3D brain MRI at 0.055 tesla

**DOI:** 10.1126/sciadv.adi9327

**Published:** 2023-09-22

**Authors:** Christopher Man, Vick Lau, Shi Su, Yujiao Zhao, Linfang Xiao, Ye Ding, Gilberto K. K. Leung, Alex T. L. Leong, Ed X. Wu

**Affiliations:** ^1^ Laboratory of Biomedical Imaging and Signal Processing, The University of Hong Kong, Hong Kong SAR, People’s Republic of China.; ^2^ Department of Electrical and Electronic Engineering, The University of Hong Kong, Hong Kong SAR, People’s Republic of China.; ^3^ Department of Surgery, LKS Faculty of Medicine, The University of Hong Kong, Hong Kong SAR, People’s Republic of China.

## Abstract

In recent years, there has been an intensive development of portable ultralow-field magnetic resonance imaging (MRI) for low-cost, shielding-free, and point-of-care applications. However, its quality is poor and scan time is long. We propose a fast acquisition and deep learning reconstruction framework to accelerate brain MRI at 0.055 tesla. The acquisition consists of a single average three-dimensional (3D) encoding with 2D partial Fourier sampling, reducing the scan time of T1- and T2-weighted imaging protocols to 2.5 and 3.2 minutes, respectively. The 3D deep learning leverages the homogeneous brain anatomy available in high-field human brain data to enhance image quality, reduce artifacts and noise, and improve spatial resolution to synthetic 1.5-mm isotropic resolution. Our method successfully overcomes low-signal barrier, reconstructing fine anatomical structures that are reproducible within subjects and consistent across two protocols. It enables fast and quality whole-brain MRI at 0.055 tesla, with potential for widespread biomedical applications.

## Teaser

Large-scale 3T MRI data enables fast whole-brain scanning at 0.055T via deep learning super-resolution and image reconstruction.

## INTRODUCTION

Magnetic resonance imaging (MRI) is a highly valuable imaging modality in modern health care due to its noninvasive, nonionizing, fundamentally three-dimensional (3D), quantitative, and multiparametric nature (*1*). Over the past five decades, the focus has been on high-field MRI (1.5 and 3 T), which are now routinely used for diagnosing diseases/injuries as well as many other biomedical applications (*2*–*4*). However, high-field MRI scanners are scarce and unevenly distributed worldwide. They are predominantly found in specialized radiology departments and centralized imaging centers in developed countries but are rarely available in low- to middle-income countries. For example, there are only 0.7 MRI scanners per million inhabitants in Africa, compared to 40 and 55 per million in the United States and Japan, respectively (*5*, *6*). These scenarios mostly arise from the high installation, maintenance, and operational costs required to house high-field superconducting MRI scanners in shielded rooms and to operate them with specialized radiographic technicians. Consequently, these scanners are hardly accessible to trauma centers, acute care facilities, pediatric clinics, and community clinics. Low- to middle-income countries, which constitute approximately 70% of the world’s population, have limited or no access to them (*7*), highlighting a growing disparity in MRI accessibility in health care (*5*).

In recent years, there has been intensive development of low-cost MRI scanners at ultralow-field (ULF) strengths (<0.1 T) for portable and point-of-care clinical applications (*8*–*14*). Several studies have demonstrated the successful implementation of key neuroimaging protocols on ULF scanners, providing clinically valuable information for diagnosing brain lesions, such as tumors and strokes (*9*, *11*, *15*–*17*). The need for radio-frequency (rf) shielding rooms has also been eliminated through active detection and removal of external electromagnetic interference signals using analytical and deep learning (DL) methods (*11*, *15*, *18*, *19*). These developments have paved the way for true point-of-care applications of shielding-free ULF MRI scanners, for example, in intensive care units and coronavirus disease 2019 wards (*11*, *15*–*17*).

However, the markedly lower signal-to-noise ratio (SNR) at ULF compared to that at high field undermines its clinical value and hinders its widespread clinical adoption (*11*, *15*–*17*). This issue intrinsically stems from the physics that the magnetic resonance (MR) signal is proportional to the field strength *B*_0_^2^, while SNR scales with *B*_0_^7/4^ approximately at low field; therefore, the MR signal at ULF is orders of magnitude weaker than that at 3 T (*20*–*22*). The issue is exacerbated by the fact that almost all ULF MRI developments so far directly adopt the traditional analytical image reconstruction methodologies from the high-field MRI, although several groups have attempted to deploy alternative computational methods such as compressed sensing and MR fingerprinting for ULF image reconstruction (*23*, *24*). Consequently, the usefulness of ULF MRI is severely compromised because of its long scan time, low image SNR, low spatial resolution, and hardware-related artifacts. Note that typical MRI resolution is sometimes anisotropic and that scanning multiple orientations is often required in clinical MRI, further lengthening the total scan time. To overcome these limitations, it is imperative to explore different approach to image formation to advance ULF MRI speed and quality.

DL powers a paradigm shift and has shown promise in various high-field MR image reconstruction tasks, including artifact reduction, denoising, and reconstruction from undersampled k-space data (*25*–*30*). This derives from the exceptional feature extraction capability of deep neural network from historical data. Alongside the increasing availability of large-scale, high-quality, and high-field human MRI data, e.g., from Human Connectome Project (HCP) Consortium and U.K. Biobank (*31*–*34*), DL is expected to escalate the development of ULF MRI. Several earlier attempts have been made to reconstruct ULF images via DL (*35*–*37*). For example, one strategy is to exploit the omnipresent 3D structural features shared across humans for all organs including the brain. They arise from genetically predefined human anatomy and present as a broad range of 3D structural characteristics in MR images of various contrasts acquired with different imaging protocols. Recently, one study reported a DL superresolution (SR) approach to synthesize 1-mm isotropic T1-weighted (T1W) MPRAGE-like 0.064 T brain images from anisotropic and coregistered T1W and T2W image datasets (*38*). However, this method is not of general purpose. The acquisition of the required T1W and T2W datasets is also time-consuming. More recently, we proposed a dual-acquisition 3D DL SR approach to directly leverage the homogeneous brain anatomy available in high-field human brain MRI data (*39*). It boosted the spatial resolution of 3-mm isotropic 0.055 T MRI T1W and T2W images to isotropic 1.5 mm and suppressed noise and artifacts. However, the acquisition of these original 3-mm 3D datasets remains slow, 8.6 and 11.2 min for T1W and T2W, respectively.

In this study, we aim to advance brain ULF MRI for speed and quality via DL. We achieve fast 3D brain MRI at 0.055 T by directly integrating the accelerated MR data acquisition and the DL 3D image formation that reconstructs images from incomplete 3D k-space data with SR. Specifically, we reduced the acquisition time of the 3-mm isotropic T1W and T2W 0.055 T brain datasets to 2.5 and 3.2 min, respectively. It was implemented on a custom-built shielding-free 0.055 T MRI head scanner (*11*) by acquiring a single number of excitation (NEX) with incomplete 2D partial Fourier (PF) k-space sampling. For reconstruction, we designed and used an end-to-end fully 3D DL model, which was trained on synthetic 3D ULF data simulated from publicly available large-scale T1W and T2W 3 T brain data from HCP. We applied the model to the undersampled low-resolution experimental 0.055 T data from healthy and elderly volunteers. The results show that the proposed acquisition and DL reconstruction can achieve fast 3D T1W and T2W whole-brain imaging with effective reduction of artifacts, and noise, and increase in spatial resolution at ULF.

## RESULTS

### Fast 3D data acquisition protocol at 0.055 T for brain MRI

We implemented and optimized two common neuroimaging protocols, i.e., T1W and T2W imaging, on a 0.055 T MRI head scanner developed in our laboratory (*11*). It operates using a standard alternating current (ac) power outlet and is low cost to build (with hardware material costs under US$20,000 for quantity production). Using a permanent samarium-cobalt magnet and DL for cancellation of electromagnetic interference, it requires neither magnetic nor rf shielding cages. A 3D fast spin echo (FSE) sequence was used to increase SNR in comparison to 2D sequence, exploiting the short T1 values of brain tissues at ULF for efficient whole-brain data acquisition (*11*, *22*, *40*). The spatial resolution was 3 mm isotropic by acquisition to represent the continuous 3D brain structures and to facilitate the effective extraction of 3D features by the 3D DL model. Data were acquired with single NEX with 2D PF sampling of a fraction of 0.7 in each of the two phase-encoding (PE) directions, i.e., left-right (LR) and superior-inferior (SI) directions. 2D elliptical PE sampling pattern was also incorporated. The ULF MRI scan time was 2.5 and 3.2 min for T1W and T2W protocols, respectively. Details of parameters of the two T1W and T2W protocols are provided in table S1.

### PF-SR DL reconstruction architecture

**Fig. 1.**
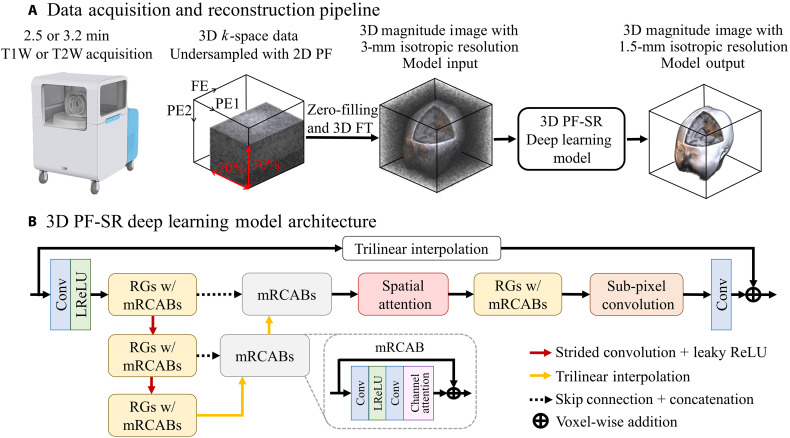
Data acquisition and DL reconstruction pipeline for fast ULF isotropic 3D brain MRI at 0.055 T. (**A**) Unlike typical ULF scans that acquire multiple NEXs, the proposed acquisition scheme acquires a single NEX, together with 2D PF sampling of a PF fraction of 0.7 in each of the two PE directions, in the FSE protocols for both T1W and T2W contrasts. The scan time is 2.5 and 3.2 min for T1W and T2W contrasts, respectively. The 3D data are then reconstructed by an end-to-end 3D DL model, which learns a residue between the high-resolution image and the interpolated low-resolution image. (**B**) The overall architecture of the DL 3D PF-SR reconstruction model. It is composed of residual groups (RGs) with modified residual channel attention blocks (mRCABs), multiscale feature extraction, spatial attention, and subpixel convolution. 3D convolution layer allows the effective extraction of 3D brain structural features. Multiscale feature extraction learns local features at the top-scale level and semiglobal features at the middle- to bottom-scale level. Spatial attention exploits the interspatial relationships by modulating the extracted features, which are then upsampled to the high-resolution space via subpixel convolution and transformed into a 3D image residue through a convolution layer. The final 3D image is formed by voxel-wise addition of the 3D image residue and the interpolated low-resolution image.

We implemented an end-to-end fully 3D DL model for reconstruction of PF-sampled low-resolution noisy ULF data. Overall architecture of this DL PF-SR model is shown in Fig. 1. A single PF-sampled low-resolution 3D image data (3-mm isotropic resolution, low SNR, and 2D PF sampling with a fraction of 0.7) was treated as the model input. 3D convolution layers were used to extract features of the isotropic and continuous 3D structures. Multiscale feature extraction with residual group (RG), inspired by residual channel attention network (RCAN) (*41*), and modified residual channel attention block (mRCAB) allowed the extraction of multiscale high-level features. Small kernel size at the top-scale level allowed the extraction of local image features, while the increased receptive field of 3D convolution layers at the middle- to bottom-scale level enabled the learning of semiglobal image features (*42*–*44*). Channel and spatial attentions were used to modulate the high-level features based on their interchannel and interspatial relationships, respectively (*45*). The modulated features were fed into a cascade of RGs, upsampled to the high-resolution feature space using 3D subpixel convolution layer (*46*), and transformed into a high-resolution 3D image residue using 3D convolution layer (*39*). The final high-resolution 3D image output was formed from the image residue and the trilinearly upsampled model input.

### DL PF-SR model training and testing

The T1W or T2W model was trained using undersampled low-resolution 3D input image data (3-mm isotropic resolution, low SNR, and 2D PF sampling with a fraction of 0.7) and high-resolution 3D target image data (1.5-mm isotropic resolution and high SNR). We used 1248 and 1182 pairs of such 3D training data for the T1W and T2W models, respectively. These were synthesized from publicly available human brain 3 T MRI data from the HCP Consortium (*31*). Each model was tested with 200 synthetic ULF data.

**Fig. 2.**
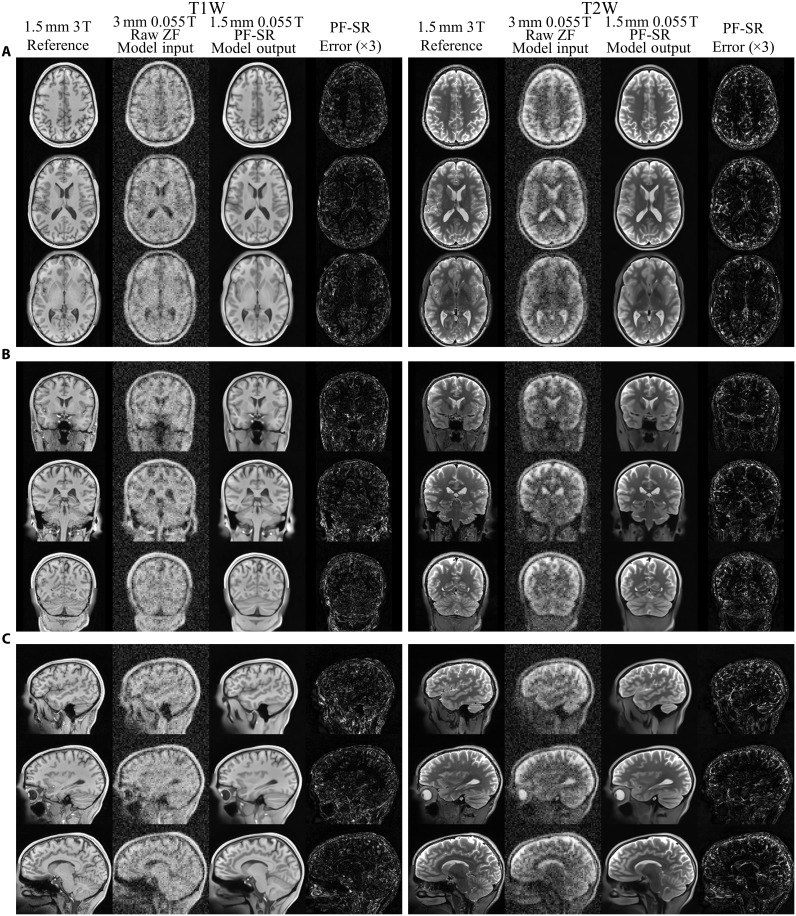
Reconstruction of synthetic low-resolution noisy 3D data with 2D PF sampling, synthesized from HCP 3-T human brain data, using the proposed PF-SR method. Two models were independently trained for T1W and T2W contrasts, respectively. (**A**) T1W and T2W axial images are shown. Model input, raw zero-filled (ZF) data, is a noisy and blurred 3D image data of 3-mm isotropic resolution with 2D PF sampling of a fraction of 0.7 along two PE directions in *k*-space (i.e., LR and SI directions). The PF-SR results and the high-field high-resolution reference have 1.5-mm isotropic resolution. The error maps (scaled by a factor of 3) with respect to the high-field reference are shown. (**B**) Coronal and (**C**) sagittal images from the same 3D data. PF-SR effectively reduced PF-related artifacts, and noise, increased spatial resolution, and recovered structures, which were consistent with the high-field reference.

**Fig. 3.**
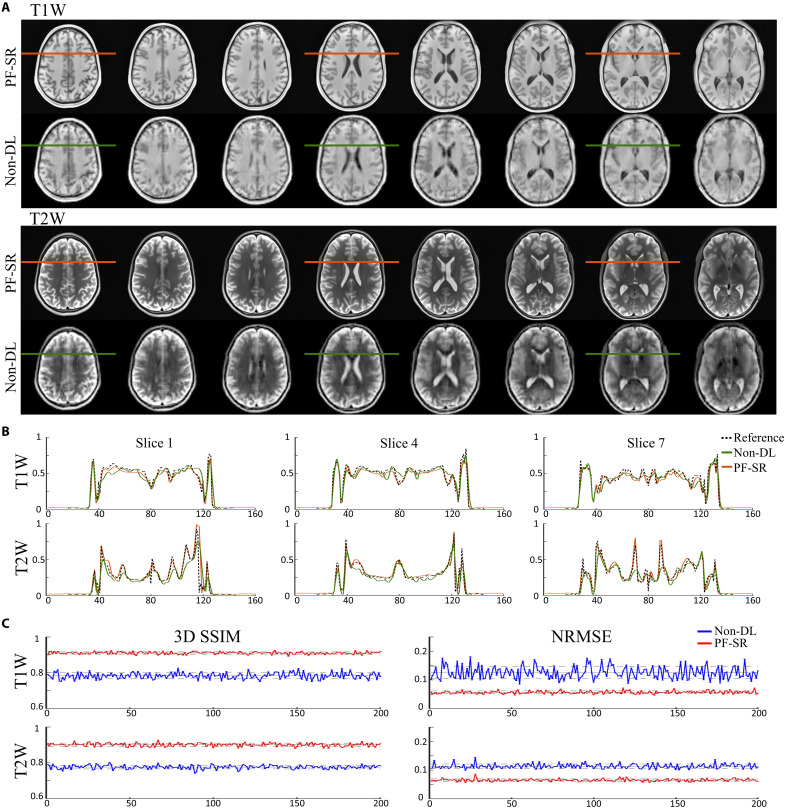
Direct comparison of traditional non-DL method and proposed PF-SR method on synthetic low-resolution noisy 3D data with 2D PF sampling, synthesized from 3-T human brain data. (**A**) Image comparison of T1W and T2W results. Non-DL consists of PF reconstruction using conventional iterative 2D projection onto convex sets (POCS), followed by block matching with 4D filtering (BM4D) 3D denoising and tricubic 2× interpolation. Eight T1W and T2W axial images are shown. PF-SR outperformed non-DL with better artifact reduction and structure restoration. (**B**) Image intensity profiles of non-DL and PF-SR, specified by the colored lines in three different slices shown in (A). PF-SR yielded profiles closer to reference profiles, indicating notably better restoration of fine structures than non-DL. (**C**) 3D structural similarity index measure (SSIM) and normalized root mean square error (NRMSE) results of non-DL and PF-SR with respect to the high-field high-resolution reference. Their SD lines are also shown. 3D SSIM and NRMSE were computed at central 60 axial slices for 200 3D synthetic testing data. PF-SR gave consistently higher 3D SSIM and lower NRMSE than non-DL.

Figure 2 shows the typical reconstruction results using the proposed PF-SR method on synthetic ULF T1W and T2W data of a healthy subject simulated from high-field data with 2D PF sampling at a fraction of 0.7 along LR and SI directions. Raw zero-filled (ZF) results are also shown for comparison. They were directly reconstructed from the PF-sampled noisy k-space data with missing data filled by zero and used as model input. PF-related blurring and ringing artifacts, as well as noise, were markedly reduced using the PF-SR. Spatial resolution was substantially enhanced, and numerous structures were recovered with high 3D fidelity in accordance with the high-resolution reference. The eight axial slices in Fig. 3 show a direct comparison of reconstruction on synthetic ULF T1W and T2W data using the traditional non-DL method and the proposed PF-SR. Non-DL consists of 2D PF reconstruction by conventional iterative projection onto convex sets (POCSs) (*47*, *48*), 3D denoising by block matching with 4D filtering (BM4D) (*49*), and tricubic interpolation for 2× SR. PF-SR consistently outperformed non-DL method at different slice locations of the brain in terms of artifact and noise reduction, as well as recovery/reconstruction of numerous anatomical structures (Fig. 3, A and B). Image intensity profile comparison and quantitative evaluation in Fig. 3C support that PF-SR consistently achieved higher 3D structural similarity index measure (SSIM) and lower normalized root mean square error (NRMSE) than non-DL method across 200 synthetic ULF T1W and T2W testing data. Figure S1 compares the reconstruction of PF-SR and our recent dual-acquisition SR (dual-SR) method. Dual-SR uses two low-resolution noisy data acquisition and with no 2D PF sampling (i.e., full sampling) to perform SR (*39*). Two methods yielded similar reconstruction performance despite the much faster data acquisition enabled by PF-SR. As demonstrated in fig. S2, PF-SR reconstruction could also recover brain lesions in synthetic ULF data of patients.

### Evaluation of DL PF-SR MRI at 0.055 T in volunteers

We recruited 15 healthy volunteers (25 to 69 years old). They were scanned using the 0.055 T MRI head scanner with scan time of 2.5 and 3.2 min for T1W and T2W data, respectively. The acquired data were reconstructed using both the proposed PF-SR method and the traditional non-DL method for direct comparison. The volunteers were also scanned using a standard clinical 3 T MRI scanner with 1.5-mm isotropic resolution for comparison.

**Fig. 4.**
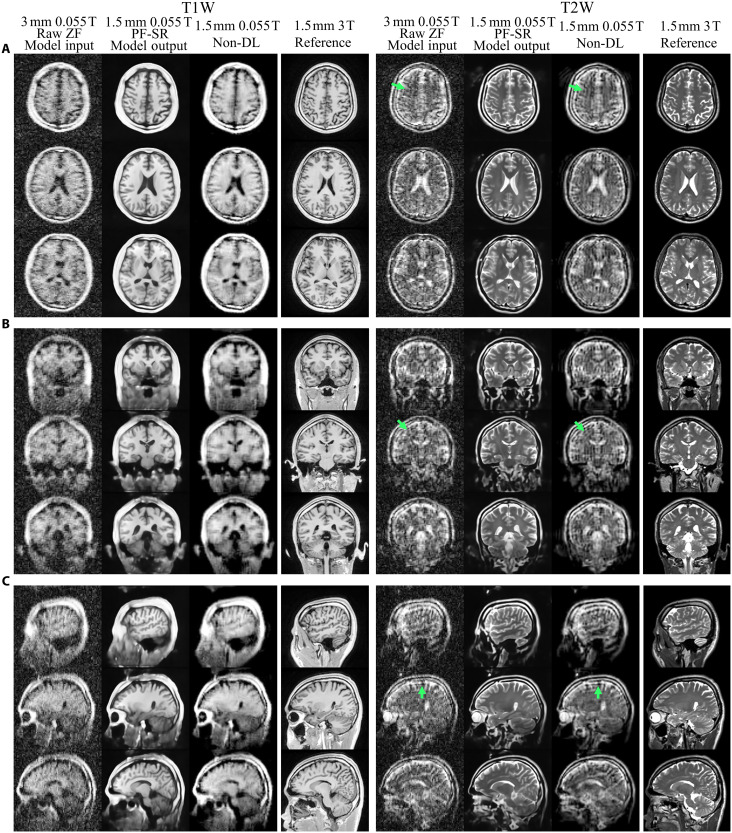
Reconstruction of experimental low-resolution 3D brain data with prospective 2D PF sampling from one healthy volunteer (33-year-old male), acquired from a low-cost shielding-free 0.055-T MRI head scanner, using traditional non-DL and PF-SR. Non-DL consists of conventional 2D POCS PF reconstruction, followed by BM4D 3D denoising and tricubic 2× interpolation. (**A**) T1W and T2W axial images are shown. Low-resolution model input is the raw 3D ZF data with 3-mm isotropic resolution and prospective 2D PF sampling along LR and SI directions. Results of non-DL and PF-SR as well as the reference images, which were acquired separately using a 3 T clinical MRI scanner from the same volunteer, have 1.5-mm isotropic resolution. (**B**) Coronal and (**C**) sagittal images from the same healthy volunteer. Ringing artifacts are clearly observable in the ZF and non-DL results (pointed by green arrows). Noticeable artifacts, and noise reduction, resolution enhancement, and recovery of structures could be achieved in both T1W and T2W images using PF-SR. Note that the 3 T reference data were coregistered to the 0.055 T data using rigid 3D translations and rotations for better visual comparison. The 0.055 T scan time was 2.5 and 3.2 min, respectively.

**Fig. 5.**
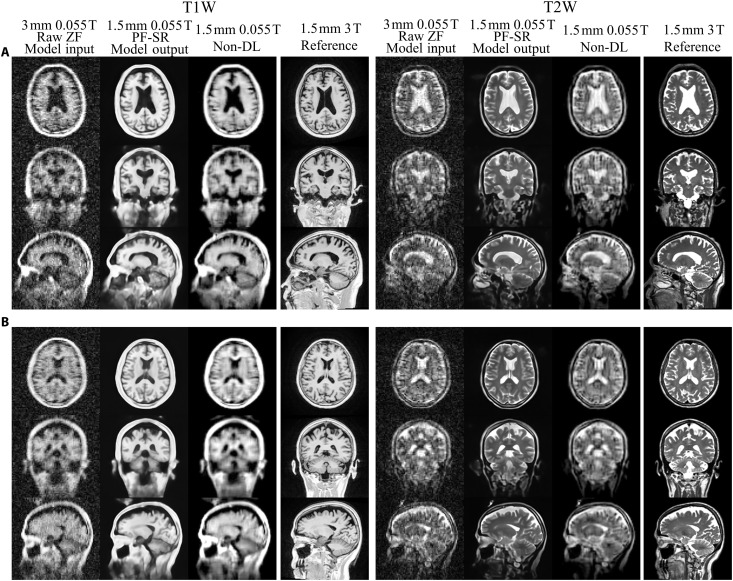
Reconstruction of experimental low-resolution 3D brain data with prospective 2D PF sampling from two elderly volunteers, acquired from a low-cost shielding-free 0.055-T MRI head scanner, using traditional non-DL and the proposed PF-SR method. (**A**) A 69-year-old male. (**B**) A 68-year-old female. T1W and T2W images are shown. Low-resolution noisy raw image is a 3D data with 3-mm isotropic resolution and prospective 2D PF sampling along LR and SI directions. Results of non-DL and PF-SR as well as the reference images, which were acquired separately using a 3 T clinical MRI scanner from the same volunteer, have 1.5-mm isotropic resolution. Aging-related brain atrophy, central ventricle enlargement, and gray/white matter shrinkage could be reliably reconstructed using PF-SR with better artifacts, and noise reduction, resolution enhancement, and recovery of structures compared to non-DL.

Typical reconstruction results of experimental ULF T1W and T2W data from one healthy volunteer are shown in Fig. 4 (see movies S1 and S2 for whole-brain results). PF-SR effectively reduced PF-related blurring and ringing artifacts, noise, and boosted spatial resolution in comparison to the non-DL method. Gray/white matter and cerebrospinal fluid (CSF) were clearly delineated in PF-SR. Structures were recovered with high clarity in PF-SR compared to non-DL and were consistent with the 3 T reference. Note that the 3 T reference from separate clinical scan was coregistered to the 0.055 T data using simple rigid 3D translations and rotations to facilitate visual comparison. Figure 5 shows the results of experimental ULF T1W and T2W data from two elderly volunteers. PF-SR could reliably depict aging-related brain atrophy, i.e., central ventricle enlargement, gray/white matter shrinkage, and peripheral CSF region expansion, in agreement with the 3 T reference, whereas non-DL results exhibited high level of noise and artifacts and loss of structural details.

**Fig. 6.**
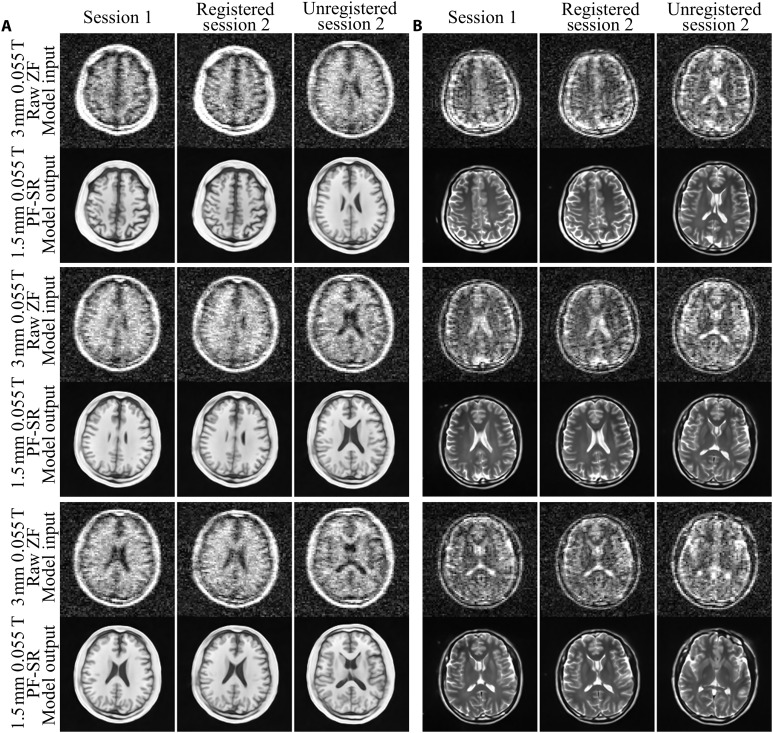
Experimental inter-exam reproducibility from two 0.055-T imaging exam sessions from the same volunteer with randomly different head positions using PF-SR. (**A**) T1W images from a 33-year-old male. (**B**) T2W images from a 27-year-old male. PF-SR results among two imaging exam sessions were coregistered through rigid 3D translations and rotations to match their positions to foster visual comparison. Anatomical structures reconstructed by PF-SR were found to be highly consistent among the two exam sessions, demonstrating the reproducibility and robustness of PF-SR on recovering structures.

Experimental inter-exam reproducibility test on T1W and T2W imaging was performed to evaluate the reconstruction consistency of PF-SR from the same volunteer with different head positions during two different exam sessions (Fig. 6). Here, PF-SR results from two sessions were coregistered using simple rigid 3D translations and rotations for visual comparison. Afterward, brain anatomical structures were observed to be highly consistent among the two sessions. Together with the high intra-exam reproducibility (fig. S3), these results demonstrate the robustness of PF-SR in reconstructing 3D brain anatomical structures. Figure S4 illustrates the effect of training sample size on reconstruction performance of PF-SR on synthetic and experimental ULF data. Noticeable loss of details was found in results of PF-SR with smaller, particularly in 25%, training sample size, indicating the importance of adequately large training sample size on reconstruction.

## DISCUSSION

In this study, we achieved fast and high-quality 3D whole-brain MRI at 0.055 T through an integrated PF sampling acquisition and DL reconstruction framework. Fast 3D imaging was realized by single-NEX acquisition with 2D PF sampling of a fraction of 0.7 along each of the two PE directions. This scheme shortened the scan time of the 3-mm isotropic ULF T1W and T2W whole-brain imaging protocols to 2.5 and 3.2 min, respectively. The 3D DL PF-SR reconstruction model was trained on synthetic ULF data simulated from large-scale 3 T human brain MRI data. We demonstrated its effective reduction of PF-related blurring and ringing artifacts, noise, and enhancement of spatial resolution to synthesized or synthetic 1.5-mm isotropic resolution in both synthetic and experimental ULF results.

### Accelerating 3D ULF T1W and T2W imaging protocols via 2D PF sampling

MR signal at ULF, e.g., 0.055 T, is approximately three orders of magnitude weaker than that at 3 T (*22*), resulting in marked SNR reduction at ULF. Existing ULF imaging protocols acquire and average multiple NEXs to increase SNR (*8*–*11*, *50*), but this lengthens data acquisition time. Our recent DL SR method on ULF adopted a dual-acquisition strategy to achieve quality 3D ULF T1W and T2W data with synthetic 1.5-mm isotropic resolution via a tailored 3D DL SR model (*39*). The scan time of the corresponding 3-mm isotropic T1W and T2W data was 8.6 and 11.2 min, respectively. Such long scans are highly vulnerable to intra-scan subject motions and cause discomfort because subject is required to remain still during scanning. In this study, we succeeded in reducing the acquisition time of 3-mm isotropic T1W and T2W data to 2.5 and 3.2 min, respectively. This was achieved by single-NEX acquisition combined with 2D PF sampling. The resulting fast acquisition is expected to lead to better MRI experience and is suitable for subjects who cannot withstand long scan times.

### PF-SR reconstruction via 3D DL for noise and artifact reduction

We implemented the 3D PF-SR model and trained it on synthetic ULF data simulated from large-scale 3 T human brain MRI data. The synthetic and experimental ULF results demonstrated that the model can effectively reconstruct from incomplete PF k-space data and suppress the high level of artifacts and noise that are intrinsic to ULF MRI using traditional analytical reconstruction methods. PF-SR reconstruction also notably increases spatial resolution via SR. These desirable features arise from the three characteristics of the model: (i) isotropic 3D convolution layers, (ii) multiscale feature extraction, and (iii) channel and spatial attention. All convolution layers and the synthetic ULF model training data are isotropic 3D to maximally facilitate the extraction of the 3D brain structural features from the large-scale 3 T human brain data. Small kernel size at the top-scale level of multiscale feature extraction enables the model to learn local features, e.g., blurring artifact, while the middle to the bottom-scale level allows the extraction of semiglobal feature, e.g., ringing artifact, by increasing the receptive field of convolution layer (*42*–*44*). Thus, a single 3D PF-SR model is able to effectively learn both local and semiglobal features. Channel and spatial attention exploits the interchannel and interspatial relationships among the extracted multiscale features. Note that no intensity correction of different brain tissues (*51*) was performed to the synthetic ULF model training data, and the trained models can readily reconstruct experimental ULF data. This is because we have experimentally optimized both T1W and T2W ULF protocols so that the T1W and T2W contrasts of various brain tissues, such as the gray/white matter and CSF, in the ULF data are similar to those in the high-field.

**Fig. 7.**
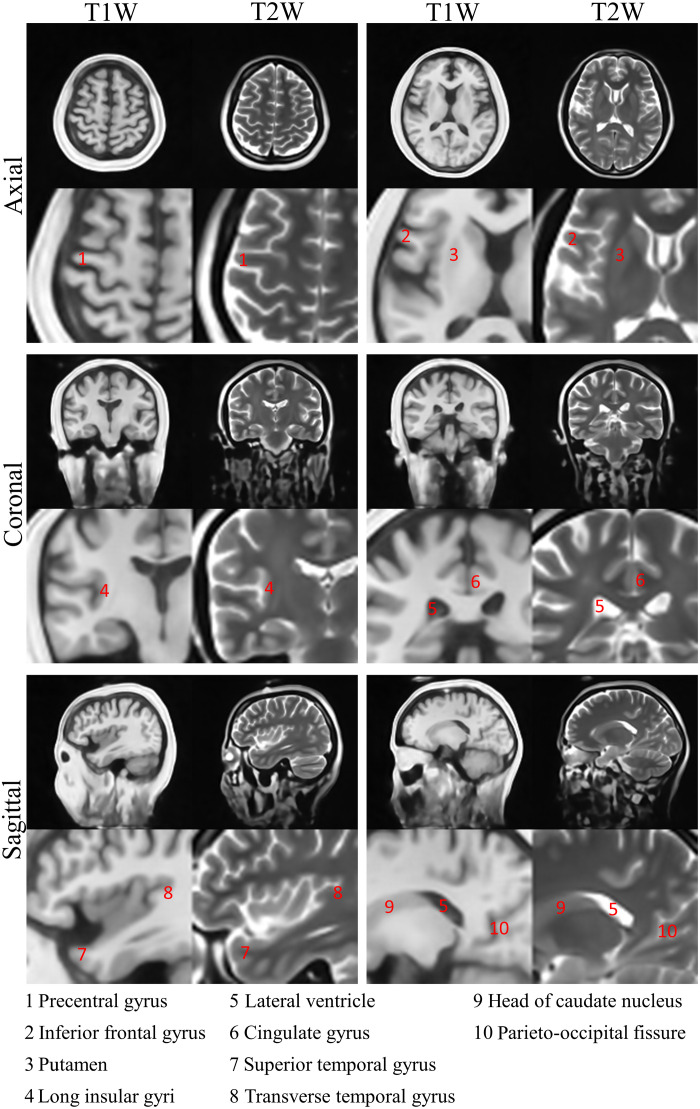
Experimental inter-contrast consistency from one healthy volunteer (40-year-old female) using PF-SR. Anatomical structures by PF-SR between T1W and T2W images are consistent and highly complementary in appearance as expected, indicating that the two PF-SR models, which were independently trained on T1W and T2W images separately, recovered the same brain tissue structures.

Normal brain tissues are highly complementary in appearance in T1W and T2W images. The restored structures by PF-SR in the T1W and T2W results are consistent and exhibit such complementary appearance as demonstrated in Figs. 4 and 5 together with direct comparisons shown in Fig. 7. This indicates that even two PF-SR models were trained separately on T1W and T2W images, they reveal the same tissue structures. Such inter-contrast consistency supports the superior ability of PF-SR in reconstructing brain structural details. Such high-fidelity reconstruction is further supported by the excellent inter-exam reproducibility (Fig. 6). Note that intra-exam test (i.e., performed with the same volunteer head position during repetitive scanning) also demonstrates excellent reproducibility as expected (fig. S3). No 3D rigid coregistration was done in this intra-exam test. PF-SR restored structures consistently among the three sessions, implying the insensitivity of PF-SR to high noise level on restoring structures.

We have examined the model performance on three training sample sizes (i.e., 25, 50, and 100% of the HCP training sample size) as shown in fig. S4. The lowest and least fluctuating training loss is achieved when the model is trained on 100% training sample size. The increase in training sample size allows the model to extract more general 3D brain structural features. In addition, it reduces the bias from individual training data, resulting in more stable model training. On the basis of the performance on synthetic and experimental ULF data (fig. S4, B and C), we anticipate that expanding the training sample size by including other high-field brain data may help to improve the PF-SR reconstruction. Doing so would entail complex dataset-specific preprocessing steps and more model training time.

We recognize that the model is, to a certain extent, specific to the contrast on which it is trained. This is expected because 3D brain image features depend on both brain anatomical structures and image contrasts. In this study, we train two PF-SR models separately and independently for T1W and T2W data. It is plausible to train single model for both T1W and T2W data although it may compromise the reconstruction performance. We also observe that the model is specific to the orientation of the 3D brain data although this limitation does not really compromise its usefulness in practice.

### Fast brain ULF MRI for pathology detection

**Fig. 8.**
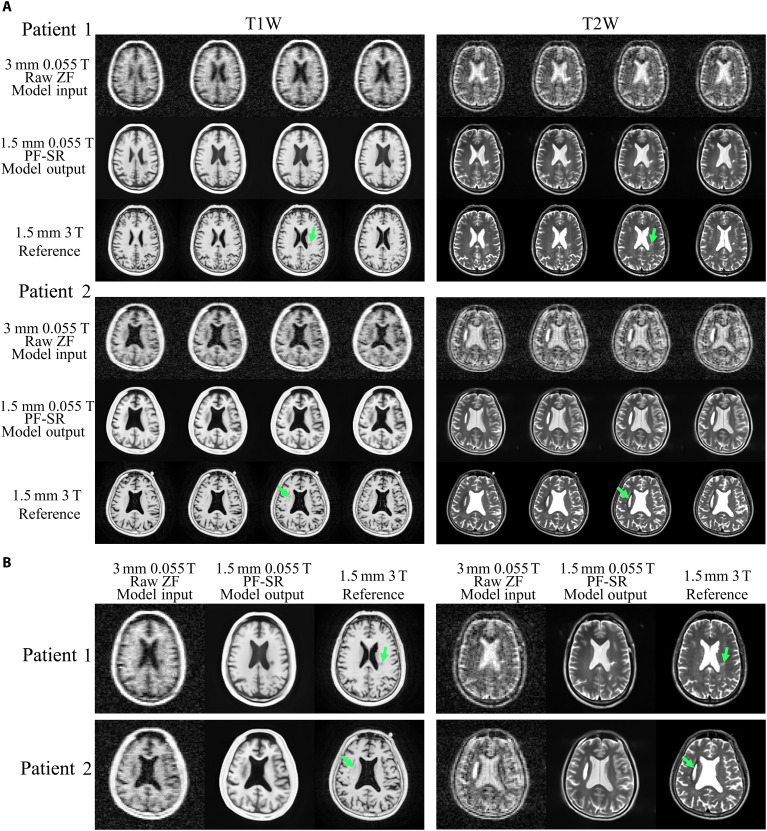
Reconstruction of experimental low-resolution 3D brain data with prospective 2D PF sampling from two patients, acquired from a low-cost shielding-free 0.055-T MRI head scanner, using PF-SR. (**A**) Patient 1 was a 64-year-old male with chronic (~10 years) ischemic stroke. Patient 2 was a 53-year-old male with right basal ganglia hematoma due to (>2 months) hemorrhage. Four axial slices from PF-sampled low-resolution noisy raw images and PF-SR results are shown for each patient, as well as the corresponding 3 T reference images. (**B**) Enlarged views of single representative slices. Locations and boundaries of lesion reconstructed by PF-SR were supported by 3 T reference images.

Results of synthetic ULF data simulated from high-field brain data containing lesions, as shown in fig. S2, together with preliminary patient results (Fig. 8), indicate that lesions could be recovered by DL PF-SR. This supports the ability of PF-SR to delineate brain lesions that differ from the normal human brain structures. Nevertheless, future study is necessary to evaluate and validate the sensitivity and specificity of the proposed PF-SR ULF MRI for lesion detection. For example, a large cohort of patients with brain lesions will be needed to carefully evaluate the reliability of the proposed fast acquisition and DL reconstruction framework for detecting different types of brain lesions with varying extents and locations. In addition, the image contrast of a specific type of lesions is subject to the MR relaxation properties, the imaging sequence type, and its contrast-related parameters. The MR relaxation properties are expected to be different between ULF and high-field given the markedly lower *B*_0_ field strength in ULF compared to high-field (*40*, *52*–*54*). This can easily result in differences in contrast for various types of lesions between ULF and high-field data. Such difference in lesion appearance can be observed in Fig. 8A when comparing 0.055 T and 3 T results, even in the raw ZF images. Therefore, it is necessary to further optimize ULF MRI protocols and their parameters to enable effective reconstruction of various types of brain lesions and to provide comparable lesion contrast to high-field MRI if high-field data are used for synthesizing training data. Alternatively, it is possible to train the PF-SR reconstruction model using datasets containing simulated lesions with diverse contrasts, locations, contours, and sizes, exposing the reconstruction model to atypical structures during the training stage.

### Fast 3D brain ULF MRI—Applications in guiding brain therapies

Although the HCP high-field brain data used in model training consist of young adults aged between 22 and 37 years old (*31*), the model effectively recovers aging-related brain atrophy features (Fig. 5). Gray/white matter and CSF volumes are common metrics used to estimate brain age (*55*) and characterize/detect certain neurodegenerative diseases (*56*, *57*), such as Alzheimer’s disease. Therefore, fast ULF MRI for 3D T1W and T2W images with 1.5-mm isotropic resolution potentially allows for volumetric quantification of various brain structures and possible detection of whole-brain structural aberrations in brain aging and neurodegenerative diseases (*58*–*60*). The low-cost portable fast 3D brain ULF MRI, as demonstrated in this study, can play an enabling role in advancing various image-guided brain therapies, such as transcranial magnetic stimulation, transcranial direct current stimulation, deep brain stimulation, and tumor ablation by rf or laser (*61*–*64*).

### Challenges

Low-cost ULF MRI aims to complement rather than compete with existing high-performance high-field clinical MRI in health care and to alleviate the MRI accessibility disparity worldwide (*5*, *6*, *12*). Despite the DL development demonstrated in this study, one key barrier to this goal remains to be the intrinsically low SNR at ULF (*11*, *39*). For example, the detailed structures in the lower brain regions, especially the fine gray and white matter structures in the cerebellum, are blurred in both synthetic (Fig. 2) and experimental ULF results (Fig. 4 and movies S1 and S2), likely due to the markedly lower image SNR in the cerebellum (arising from the low cerebellum MR signal intensity and the poor rf coil detection sensitivity in the lower brain region).

We envision various developments in hardware and DL to tackle this challenge in the future. For example, at ULF, the noise in MR signals is dominated by the rf receiver coil noise, while the sample noise is negligible (*21*). Therefore, ULF SNR can be substantially increased by cooling the rf receiver coil and rf preamplifier, which can be potentially implemented by cryogenic cooling or cryogen-free conduction cooling using cryocoolers (*65*–*67*). Litz wire has also been found particularly suitable for constructing ULF rf receiver coil (*68*–*70*). On the other hand, new computational strategies will be continuously developed to advance ULF image quality (e.g., higher spatial resolution by higher SR factor) by exploiting rapidly evolving DL algorithms and architectures as well as ever increasing computing power and large-scale human MRI data availability (*59*, *71*, *72*). Future efforts shall also encompass the experimental assessment and optimization of ULF data acquisition and DL image reconstruction to yield optimal trade-offs between image fidelity, resolution, contrast, scan time, and cost for each specific applications.

In conclusion, we present an integrated PF sampling acquisition and DL SR reconstruction framework to accelerate fast 3D whole-brain MRI with isotropic resolution at 0.055 T despite the markedly decreased MR signal. The proposed acquisition scheme successfully shortens the scan time of the 3-mm isotropic T1W and T2W data to 2.5 and 3.2 min, respectively. The proposed 3D DL reconstruction markedly reduces artifacts and noise and simultaneously increases resolution by learning from large-scale high-field human brain MRI data.

## MATERIALS AND METHODS

### 0.055-T MRI imaging protocol optimization

Experimental ULF brain data acquisitions were performed on a custom-built shielding-free 0.055 T MRI head scanner developed in our laboratory (*11*). The scanner is low-cost, low-power, and acoustically quiet during scanning. 3D T1W and T2W imaging protocols were implemented using 3D FSE sequence with and without inversion preparation, respectively. The exact 0.055 T protocol timing parameters were experimentally adjusted so that brain T1W and T2W images exhibit tissue contrasts similar to those at high field. Details of parameters of the two T1W and T2W FSE protocols are shown in table S1.

### DL reconstruction model architecture

We design a fully 3D DL model for reconstruction of PF-sampled low-resolution noisy ULF data. Figure 1 shows the overall architecture of the proposed 3D DL model. Inspired by RCAN (*41*) for 2D natural image SR, the proposed model incorporates RG with mRCAB. The proposed model contains 3D convolution layers, multiscale feature extraction, and channel attention (*42*, *44*). 3D convolution layers are used over 2D convolution layers to more effectively extract the features of the isotropic and continuous 3D brain structures. Multiscale feature extraction is realized by downsampling and upsampling of features twice via 3D convolution layers of stride 2 and trilinear interpolation of a factor of 2, respectively. At the top-scale level of the feature pyramid, the use of small kernel size allows the model to extract local features, such as the blurring artifact, which is resulted from the missing high-frequency *k*-space due to PF sampling. At the middle- to bottom-scale level, the receptive field of the 3D convolution layers is increased, enabling the model to learn semiglobal features (*42*–*44*), such as the ringing artifact caused by *k*-space truncation in PF sampling. Skip connection in the multiscale feature extraction retains the high frequency information lost during downsampling of features. Channel attention captures the interchannel relationship among the extracted features at different scales (*45*).

Following the multiscale feature extraction, multiscale spatial attention modulates the extracted multiscale features, exploiting the interspatial relationships (*45*). RG with mRCAB further refines high-level features of the 3D brain structures. Subpixel convolution layer upsamples the extracted low-resolution features to the high-resolution feature space (*46*). The final 3D convolution layer transforms the high-resolution features to the high-resolution 3D image residue, which corresponds to the model output. The global residual connection is used to learn the image residue, instead of the direct mapping between the high-resolution model output and the upsampled model input (*73*).

Each RG is composed of five mRCABs, and each mRCAB consists of two 3D convolution layers with a leaky rectified linear unit (LReLU) (*74*) in between and a channel attention at the end. All 3D convolution layers had kernel size of 3 × 3 × 3. The number of channels was 64 for all 3D convolution layers except 8 and 1 channel for channel attention and the last convolution layer, respectively. LReLU with negative slope = 0.1 was used. The model had approximately 29.7 million learnable parameters and 0.848 tera-floating-point operations per second.

### Training data preparation

A publicly available 3 T human brain MRI dataset, namely, the Wu-Minn HCP (HCP S1200) (*31*), was used. It included 3D T1W MPRAGE and T2W SPACE magnitude brain data, both with 0.7-mm isotropic resolution and from young subjects aged between 22 and 37 years old. Details of the two HCP S1200 protocols are provided in table S1.

To simulate PF-sampled low-resolution noisy 3D T1W and T2W ULF data from the corresponding 3 T data, we first used local mean to downsample the original high-resolution 3 T data to approximately 1.5-mm isotropic resolution to generate the model training target data. For model training input data, symmetric k-space truncation in all three directions was performed to further downsample the model training target data to 3-mm isotropic resolution. This accounts for the MR signal acquisition in the k-space and ensures that Gibbs ringing artifact caused by insufficient high-frequency k-space was present in the low-resolution model training input (*75*). Retrospective 2D PF sampling of a fraction of 0.7 was then applied along the two PE directions (LR and SI) in the k-space. The PF-sampled k-space was Fourier-transformed, and the resulting magnitude image was further degraded by the addition of Rician noise (*76*) to create the PF-sampled low-resolution noisy 3D model training input. The noise level was similar to those observed in the experimental raw ZF ULF brain images. Both model training input and target were normalized to the intensity range of [0, 1].

### Model training and testing

Two 3D DL models were trained with 1248 T1W and 1182 T2W data separately. Random extraction of 32 × 32 × 32 3D patch from the low-resolution input and the corresponding 64 × 64 × 64 3D patch from the high-resolution target was performed during model training. This allowed the model to focus on learning local and semiglobal, rather than gross, structural characteristics.

The model was optimized by minimizing the L1 loss using AdamW optimizer (*77*) with β_1_ = 0.9, β_2_ = 0.999, and weight decay of 0.01. The initial learning rate was 0.0001 and was reduced by a factor of 0.8 every 100 epochs. We trained the model for 350 epochs with a batch size of 8 on four Nvidia V100 graphics processing units using PyTorch 1.11 and CUDA 11.4. The training time was 30 and 30 hours for T1W and T2W model, respectively. The computing time on each T1W and T2W pair of low-resolution 3D data on a single GPU was 1.9 s.

We tested the model on two sets of brain data: (i) synthetic ULF data simulated from 3 T data and (ii) experimental ULF data. The proposed DL method, namely, PF-SR, was compared to a non-DL method, which consisted of 2D iterative POCS (*47*, *48*) for PF reconstruction, followed by BM4D (*49*) and tricubic 2× interpolation for 3D denoising and single-image SR, respectively. POCS iteratively enforced low-frequency phase information and data consistency with sampled data to reconstruct the missing data incurred by 2D PF sampling. BM4D iteratively gathered similar local 3D patches to form a 4D group and filters the group in a transform domain. The reconstructed high-resolution images and the error maps with respect to the high-resolution reference images in three orientations (i.e., axial, coronal, and sagittal) were examined for qualitative assessment. 3D SSIM (*78*) and NRMSE were calculated on 200 synthetic ULF data for quantitative evaluation. Image intensity profiles were plotted to evaluate the ability of PF-SR to restore fine details. We also compared PF-SR to our recent dual-SR method (*39*) on synthetic ULF data both qualitatively and quantitatively. Dual-SR first extracted features separately from two low-resolution noisy fully sampled data (i.e., without 2D PF sampling) and then exploited the complementary information between them in a tailored attentional fusion module. The fused features were upsampled to generate the high-resolution model output.

### Study participants

Fifteen healthy volunteers (25 to 69 years old) were recruited. Written informed consent was obtained from all volunteers in this study with the approval of the institutional review board. All recruited volunteers were scanned by the 0.055 T MRI head scanner developed in our laboratory (*11*) and a clinical GE 3 T MRI scanner (SIGNA Premier) for visual comparison. Details of the T1W and T2W protocols of the clinical 3 T scanner are provided in table S1. Whenever necessary, simple rigid 3D coregistration (FSL version 6.0.4) with 3D translations and rotations was performed on the 3 T data to match the orientations of 0.055T data for convenient visual comparison.
