# Supplementary material for: Deep learning enabled fast 3D brain MRI at 0.055 tesla
Source: Sci Adv. 2023 Sep 22;9(38):eadi9327. doi: 10.1126/sciadv.adi9327 (PMC10516503; doi:10.1126/sciadv.adi9327)
Supplement: Supplementary file 1 — Figs. S1 to S4 Table S1 Legends for movies S1 and S2 [file sciadv.adi9327_sm.pdf]

Science Advances
AAAS

Supplementary Materials for

Deep learning enabled fast 3D brain MRI at 0.055 tesla

Christopher Man *et al.*

Corresponding author: Ed X. Wu, ewu@eee.hku.hk

Sci. Adv. **9**, eadi9327 (2023)
DOI: 10.1126/sciadv.adi9327

The PDF file includes:

Figs. S1 to S4
Table S1
Legends for movies S1 and S2

Other Supplementary Material for this manuscript includes the following:

Movies S1 and S2

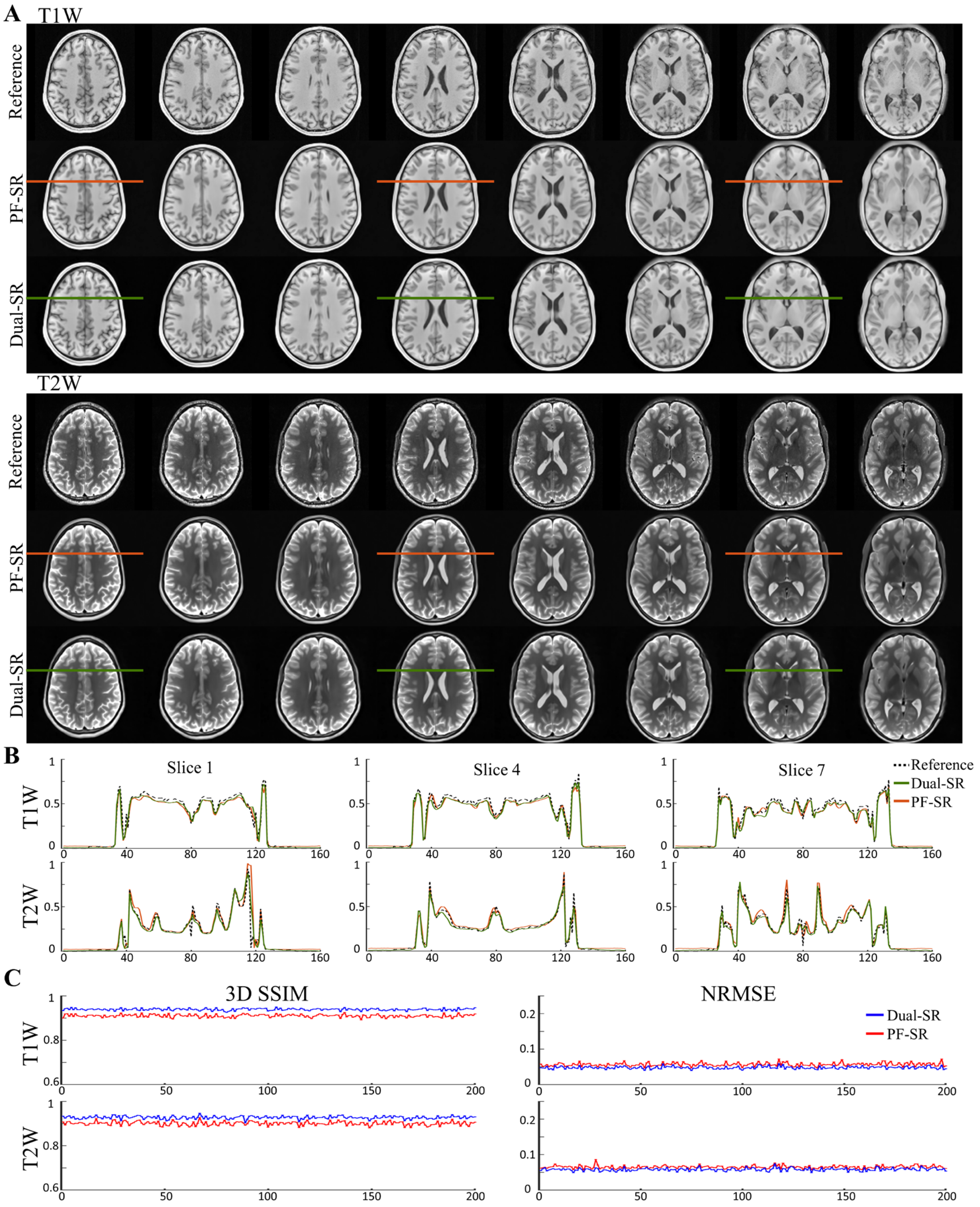

Fig. S1. Direct comparison of the proposed PF-SR method and our recent dual-acquisition SR (Dual-SR) method on synthetic low-resolution noisy 3D data, synthesized from 3T human brain data. (**A**) Image comparison of PF-SR and Dual-SR results. Dual-SR utilizes two fully-sampled acquisitions (with no 2D PF sampling) as input, while PF-SR uses a single acquisition with 2D PF sampling. Eight T1W and T2W axial images are shown. (**B**) Image intensity profiles of Dual-SR and PF-SR, specified by the colored lines in three different slices shown in (A). (**C**) 3D SSIM and NRMSE results of PF-SR and Dual-SR with respect to reference. 3D SSIM and NRMSE were computed at central 60 axial slices for 200 3D synthetic testing data. Two methods yielded similar performance in terms of artifact and noise reduction, and structure restoration, despite the much faster data acquisition enabled by PF-SR.

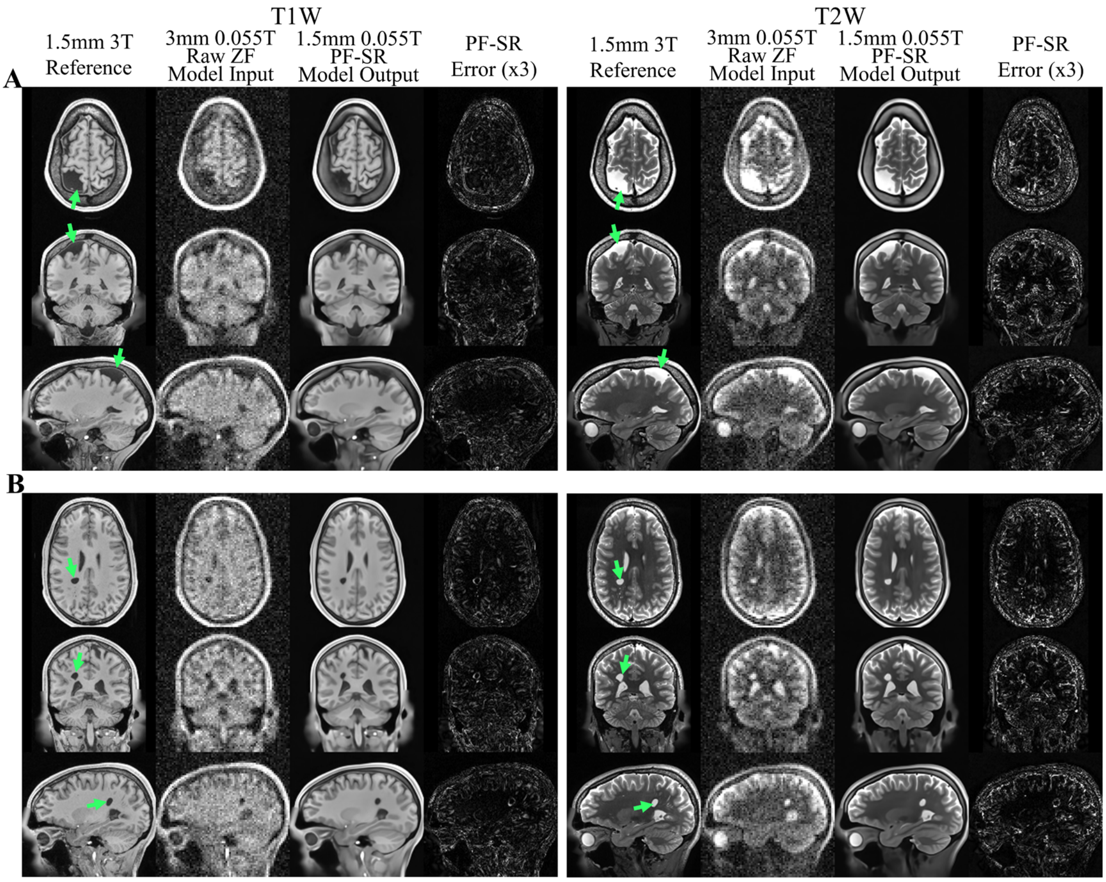

Fig. S2. Reconstruction of synthetic low-resolution noisy 3D data with 2D PF sampling, synthesized from HCP 3T human brain data with abnormal structures, using PF-SR. (**A**) Subject with dorsal arachnoid cyst. (**B**) Subject with parietal lobe cyst. Low-resolution noisy raw zero-filled (ZF) 3D data with 2D PF sampling, PF-SR results, and high-field high-resolution reference are shown. Error maps with respect to the reference are scaled by a factor of 3. Lesions could be reliably reconstructed in both T1W and T2W images using PF-SR.

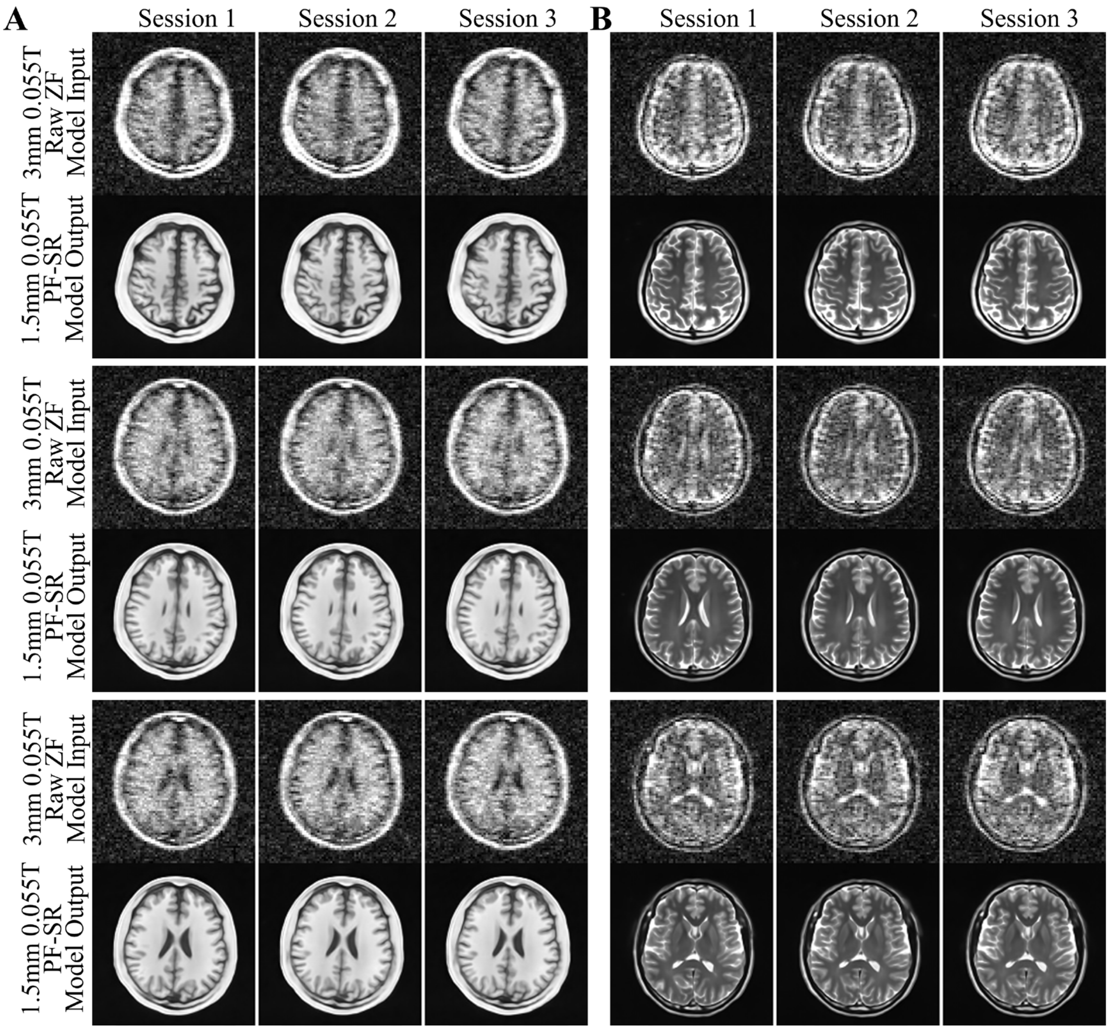

Fig. S3. Experimental intra-exam reproducibility from repetitive 0.055T scanning of the same volunteer with fixed head position using PF-SR. (**A**) T1W images from 33-year-old male. (**B**) T2W images from 27-year-old male. No 3D rigid co-registration was used in this test. Anatomical structures by PF-SR were observed to be highly consistent, indicating the stability of PF-SR reconstruction.

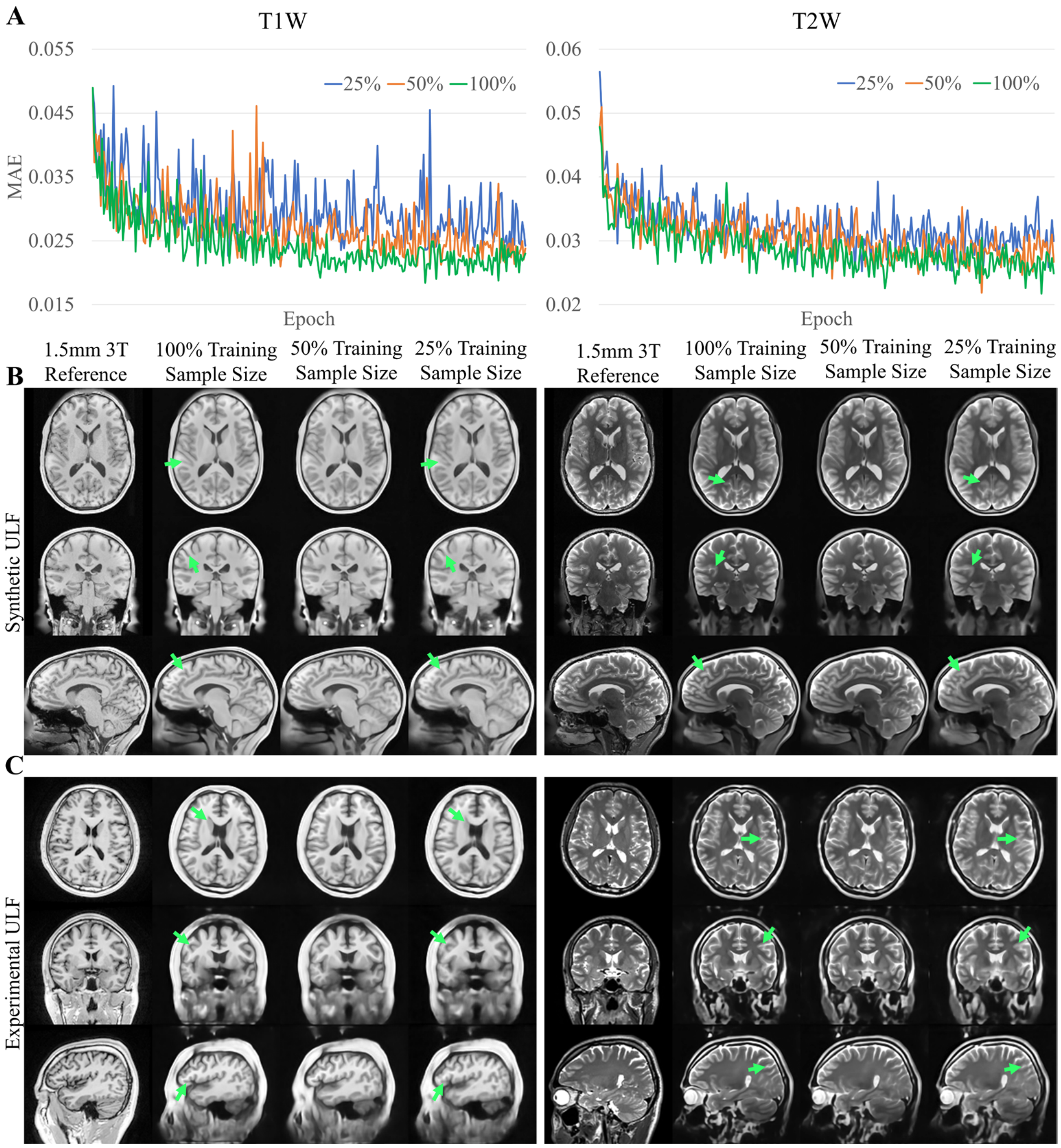

Fig. S4. Reconstruction performance of PF-SR trained with different training sample size. (**A**) Convergence plot of models trained with different sample size. 100% training sample size, which was used in this study, achieved the lowest training loss with the least fluctuation. Reconstruction of (**B**) synthetic and (**C**) experimental low-resolution noisy 0.055T 3D brain data with 2D PF sampling, using PF-SR models trained with different sample size. A typical T1W and T2W slice from each orientation is shown. More structural details were restored using PF-SR trained on 100% sample size than on 50% sample size. Apparent loss and smearing of details (pointed by green arrows) could be observed in PF-SR trained on 25% sample size.

	T1W			T2W		
	HCP	0.055T	3T Reference	HCP	0.055T	3T Reference
Sequence	3D MP-RAGE	3D FSE	3D MP-RAGE	3D SPACE	3D FSE	3D SPACE
TR (ms)	2400	590	1800	3200	1500	3000
TE (ms)	2.1	14	2	565	207	68.4
TI (ms)	1000	190	900	n/a	n/a	n/a
Echo Train Length	n/a	10	n/a	314	21	130
Flip Angle (^{o})	8	n/a	8	n/a	n/a	n/a
Bandwidth (Hz/pixel)	210	125	390	744	78	780
Matrix size	320*320*256	80*80*72	160*160*160	320*320*256	80*80*72	160*160*160
Resolution (mm)	0.7	3	1.5	0.7	3	1.5
NEX	2	1	1	2	1	1
Scan Time (mins)	7.7	2.5	3.6	8.3	3.2	2.6

Table S1. Summary of imaging parameters of T1W and T2W protocols from Human Connectome Project (HCP), 0.055T shielding-free MRI head scanner, and 3T clinical scanner. (n/a: not applicable)

Supplementary Movies

Movie S1. PF-SR reconstruction of T1W images from the same subject as in **Fig. 4**.

Movie S2. PF-SR reconstruction of T2W images from the same subject as in **Fig. 4**.
